# Somatic *TP53* Mutations Are Detectable in Circulating Tumor DNA from Children with Anaplastic Wilms Tumors^1^^2^

**DOI:** 10.1016/j.tranon.2018.08.006

**Published:** 2018-08-29

**Authors:** Taryn D. Treger, Tasnim Chagtai, Robert Butcher, George D. Cresswell, Reem Al-Saadi, Jesper Brok, Richard D. Williams, Chrissy Roberts, Nicholas M. Luscombe, Kathy Pritchard Jones, William Mifsud

**Affiliations:** * UCL Great Ormond Street Institute of Child Health, London, UK; † Francis Crick Institute, London, UK; ‡ Clinical Research Department, Faculty of Infectious and Tropical Diseases, London School of Hygiene & Tropical Medicine, London, UK; § Department of Paediatric Haematology and Oncology, Rigshospitalet, Copenhagen University Hospital, Denmark; ¶ UCL Genetics Institute, Department of Genetics, Evolution & Environment, University College London, UK; # Department of Paediatric Haematology and Oncology, Great Ormond Street Hospital, London, UK; ** Department of Histopathology, Great Ormond Street Hospital, London, UK

## Abstract

*BACKGROUND:* Diffuse anaplastic Wilms tumor (DAWT) is a rare, high-risk subtype that is often missed on diagnostic needle biopsy. Somatic mutations in *TP53* are associated with the development of anaplasia and with poorer survival, particularly in advanced-stage disease. Early identification of DAWT harboring *TP53* abnormalities could improve risk stratification of initial therapy and monitoring for recurrence. *METHODS:* Droplet digital polymerase chain reaction (ddPCR) was used to evaluate 21 samples from 4 patients with DAWT. For each patient, we assessed *TP53* status in frozen tumor, matched germline DNA, and circulating tumor DNA (ctDNA) from plasma, serum, and urine collected throughout treatment. *RESULTS*: Mutant *TP53* was detectable in ctDNA from plasma and serum in all patients. We did not detect variant *TP53* in the same volume (200 μl) of urine. One patient displayed heterogeneity of *TP53* in the tumor despite both histological sections displaying anaplasia. Concentration of ctDNA from plasma/serum taken prenephrectomy varied significantly between patients, ranging from 0.44 (0.05-0.90) to 125.25 (109.75-140.25) copies/μl. We observed variation in ctDNA throughout treatment, and in all but one patient, ctDNA levels fell significantly following nephrectomy. *CONCLUSION:* We demonstrate for the first time that ddPCR is an effective method for detection of mutant *TP53* in ctDNA from children with DAWT even when there is intratumoral somatic heterogeneity. This should be further explored in a larger cohort of patients, as early detection of circulating variant *TP53* may have significant clinical impact on future risk stratification and surveillance.

## Introduction

Wilms tumor (WT) or nephroblastoma is the most common childhood renal cancer, with 1 in 100,000 children diagnosed annually [1]. In Europe, children are treated with neoadjuvant chemotherapy prior to surgery as per The International Society of Pediatric Oncology Renal Tumors Study Group (SIOP RTSG) guidelines [2]. Conversely, in North America, children undergo immediate surgery prior to chemotherapy in accordance with the Children's Oncology Group (COG). For both groups, tumor histology and stage dictate the intensity of postoperative treatment, with chemotherapy and sometimes radiotherapy. Regardless of the protocol used, overall survival approaches 90% [3].

Despite this excellent prognosis, approximately 15% of patients relapse, and for the subgroup displaying high-risk histology, more than one in four patients need intensified treatment for disease recurrence [4]. Diffuse anaplasia (DAWT) is classified as high risk by both SIOP RTSG and COG. Needle biopsy rarely captures anaplasia, but it is found in 5%-10% of cases following surgical resection [5]. Approximately 60% of DAWTs have somatic mutations in the tumor suppressor gene *TP53* and/or 17p loss. Although these mutations likely confer an increased risk of both relapse and mortality, particularly in advanced tumor stage, genetic testing of surgically resected tumors is not routinely performed [6], [7].

At diagnosis, there are no radiological findings to clearly differentiate a WT from other renal tumors or to differentiate the subtype of WT. In most countries that follow the SIOP approach, preoperative chemotherapy is commenced without a confirmatory biopsy. Furthermore, biopsy provides limited diagnostic information in part due to the high degree of genetic intratumoral heterogeneity (ITH) found in WT [8], [9]. In order to circumvent the issues of ITH and the limitations and risks related to the biopsy procedure, minimally invasive molecular biomarkers are urgently needed.

Circulating tumor DNA (ctDNA), shed by tumor cells and detectable in a range of bodily fluids, is a promising candidate as it represents contributions from multiple tumor subclones [10]. However, efforts to characterize ctDNA in pediatric tumors have lagged behind the work achieved in adult cancers. One principal limitation in detection of ctDNA is its variable mutant allele fraction (MAF), among an abundant background of cell-free DNA released by nontumor cells. Droplet digital PCR (ddPCR) is an ideal tool for characterizing ctDNA as it provides a limit of detection comparable to MAF. This technique partitions a sample into 15,000-20,000 droplets, with PCR occurring in each droplet [11]. The number of DNA templates within each droplet is modeled by a Poisson distribution, and by quantifying the fraction of positive droplets, absolute DNA levels can be determined without extrapolation from a standard curve [12]. Due to the relative concentrations of targets and inhibitors in each droplet, ddPCR is more resistant to inhibition and more reproducible at low target concentrations than real-time quantitative PCR, making it attractive for diagnostic applications [13].

The purpose of this study was to test the feasibility of ctDNA detection in children with high-risk DAWT using plasma, serum, and urine collected throughout treatment.

## Materials and Methods

### Patients

All patients with a diagnosis of WT were enrolled in the UK-wide Improving Population Outcomes for Renal Tumours of Childhood (IMPORT) study. Informed consent to undertake genetic testing of samples was obtained as part of the study, which was approved by the national research ethics committee (London Bridge REC 12/LO/0101). Normal kidney and multisampled tumor tissues were collected at surgery, while blood and urine were collected at up to five treatment time points: diagnosis, midchemotherapy, preoperative, postoperative, and end of treatment. Patients were treated with preoperative chemotherapy regimens according to stage as per SIOP WT 2001. Nonmetastatic cases (stages I-III) received vincristine/actinomycin-D for 4 weeks, with doxorubicin added for metastatic disease (stage IV) for 6 weeks. Following nephrectomy, patients with focal or diffuse anaplasia were risk stratified to intermediate- or high-risk subgroups, respectively, and postoperative treatment was further refined according to stage of disease at surgery.

### DNA Extraction

All samples used for tumor DNA extraction were fresh-frozen specimens obtained at nephrectomy and stored at −80°C. Specimens underwent centralized histology review to confirm stage and histology, and those with tumor content of more than 50% were utilized for the study [14]. Tumor DNA and germline DNA from normal tissue were extracted by standard methods using either a standard detergent lysis/phenol-chloroform technique or the DNAeasy Blood and Tissue Kit (Qiagen). Germline DNA from cell fraction was extracted with the QIAamp DNA Blood Midi Kit (Qiagen). DNA was stored at −20°C. Extraction of ctDNA from 200 μl of plasma, serum, and urine supernatant was carried out with the Plasma/Serum Cell-free Circulating DNA Purification Mini Kit (Norgen) and the Circulating Nucleic Acid Kit (Qiagen) as per protocol. Elution volumes were 50 μl and 75 μl for the Norgen and Qiagen kits, respectively. Quantification and further quality control of tumor and germline DNA were undertaken with a NanoDrop spectrophotometer (Thermo Fisher Scientific), Qubit fluorometer (Thermo Fisher Scientific), and agarose gel.

### Sequencing of Tumor and Germline DNA

For *TP53* mutational analysis, Sanger sequencing of tumor DNA was carried out by Great Ormond Street Hospital Genetics. Bidirectional sequencing was undertaken for all 11 exons of the gene. For the five patients with *TP53* mutations, we performed Sanger sequencing on germline DNA. PCR amplification products were run on an agarose gel prior to clean-up with Illustra ExoStar 1-Step (GE Healthcare Life Sciences). Cleaned PCR products were sequenced using the BigDye Terminator v3.1 Cycle Sequencing Kit (Applied Biosystems) on an ABI PRISM 3130 Genetic Analyzer (Applied Biosystems). Sequences were examined both manually and electronically using ApE (v2.0.49 Wayne Davis, University of Utah) and 4peaks (v1.8 Mekentosi, Amsterdam) prior to genome alignment to GRCh37 with Blat [13] and functionally annotated with Annovar [14]. Single nucleotide polymorphisms (SNPs) were excluded if present in The Database of Single Nucleotide Polymorphisms (dbSNP) [15].

### ddPCR

In four out of the five cases with *TP53* mutations, assays with sequence-specific primers and TaqMan-based probes for mutant and wild-type alleles were purchased (PrimePCR ddPCR Mutation Assay Bio-Rad for *TP53*). Primer and probe sequences are not provided by Bio-Rad, but amplicon context sequence and length for each assay are as follows: p.R273C 17:7,577,060-7,577,182 65 nt; p.R337C 17:7,573,957-7,574,079 79 nt; p.R248Q 17:7,577,477-7,577,599 62 nt; p.H197L 17:7,578,333-7,578,455 65 nt. To assess optimum annealing temperature, each assay was run on a temperature gradient with germline DNA and a 50:50 mix of tumor DNA spiked into germline DNA. Each ddPCR mixture was made up of 11 μl 2× ddPCR Supermix (No dUTP; Bio-Rad), 1.1 μl mutant-specific FAM probe, 1.1 μl wild-type–specific HEX probe, 0.5 μl restriction enzyme (MseI or HaeIII; New England BioLabs), 0.5 μl nuclease-free water, and 8.8 μl sample DNA. Twenty microliters of ddPCR mixture was partitioned into droplets using the QX100 Droplet Generator (Bio-Rad). The thermal cycling profile was 95°C for 10 minutes, followed by 40 cycles of 94°C for 30 seconds and 52°C for 1 minute, then 1 cycle of 98°C for 10 minutes. Droplets were read on a QX100 Droplet Reader (Bio-Rad). Three technical replicates were used for each sample, as well as no template and positive controls (tumor DNA with mutant *TP53*). QuantaSoft software (version 1.3.2.0, Bio-Rad) was used to set thresholds manually by evaluating maximum separation between positive and negative clusters in control wells. Data are presented in accordance with minimum reporting guidelines for digital PCR studies [16]. Raw data were exported into R Studio and plotted with the ggplot2 package (H. Wickham. ggplot2: Elegant Graphics for Data Analysis. Springer-Verlag New York, 2009). MAF is calculated by dividing the concentration of the mutant allele by the sum of the concentrations of the mutant and wild-type alleles. Calculating the MAF normalizes the mutant allele signal against the background of wild-type alleles and represents the data as would be expected in a next-generation sequencing (NGS)–based assay.

## Results

### Patient Demographics

Ten patients from the IMPORT study were identified with WT displaying focal or diffuse anaplasia (confirmed by central histology review) and with plasma, serum, and urine collected at more than one time point. The clinicopathological information for each case is shown in Table 1. None of the five patients with wild-type *TP53* relapsed, while three of five patients with *TP53* mutations died of their disease.

**Table 1:** Clinicopathological Details for the Included Patients with Wilms Tumor, Including *TP53* Status

Case	Age at Diagnosis (Months)	Sex	Biopsy	Histology	Pathological Local Stage	Metastatic at Diagnosis	Relapse	Died of disease	*TP53* status
1	58	F	no anaplasia	DAWT	III	1	1	1	p.R248Q
2	58	F	no anaplasia	DAWT	I	0	1	1	p.E285K
3	47	F	no anaplasia	DAWT	III	0	0	0	wild type
4	53	F	no anaplasia	focal anaplasia	I	0	0	0	wild type
5	19	F	no anaplasia	DAWT	I	0	0	0	wild type
6	45	F	no anaplasia	DAWT	III	1	1	1	p.R337C
7	75	M	no anaplasia	DAWT	1	0	0	0	wild type
8	71	M	no anaplasia	DAWT	II	0	0	0	wild type
9	117	F	diffuse anaplasia	DAWT	I	1	0	0	p.R273C
10	108	F	unknown	DAWT	III	0	0	0	p.H179L

### Sanger Sequencing and ddPCR of Tumor Tissue

Sanger sequencing of tumor DNA identified *TP53* mutations in five patients. Multiple spatially distinct tumor samples from each patient were also analyzed on an NGS panel that included *TP53* as part of another experiment. The *TP53* mutation in case 10 was subclonal, while all other cases 1, 2, 6, and 9 were homogenous for mutant *TP53* (data not shown). Only specimens with anaplasia were used for analysis.

There is no single hotspot locus for mutant *TP53* in WT, and each patient had individual mutations as shown in Table 1. Matched germline DNA from cell fraction or normal kidney displayed the wild-type allele, confirming the mutations as somatic changes. Tumor samples were run as positive controls during the ddPCR experiments, and all samples had mutant *TP53* in the tumor (data not shown).

### ddPCR of ctDNA from Plasma, Serum, and Urine

Mutations in *TP53* from ctDNA were detectable from the four cases with mutation-specific Bio-Rad assays. For case 2, there was no commercially available mutation-specific assay.

Concentration varied significantly between patients (Table 2). The most abundant mutant ctDNA was found in case 6 at diagnosis, both in plasma and in serum, with a concentration of 125.25 (109.75-140.25) copies/μl and 121.00 (98.00-140.50) copies/μl, respectively. Case 6 had lung metastases at diagnosis, with progression of disease and new nodules postoperatively. The absence of detectable ctDNA postoperatively suggests that the high levels were derived from the primary tumor rather than metastatic deposits, as these were not removed during nephrectomy. All patients had detectable ctDNA prenephrectomy; we are unable to comment on chemonaive samples as, for two of the cases, we did not receive samples at diagnosis.

**Table 2:** Mean Concentration (copies/μl) of *TP53* Mutant ctDNA for Each Sample

Case Number	Time Point	Sample Type	Concentration (copies/μL)	Confidence Interval (copies/μL)
**1**	PrO	Serum	0.44	0.05-0.90
	PsO	Serum	6.63	4.93-8.15
	EoT	Urine	0.00	0.00-0.21
**6**	Dx	Plasma	125.25	109.75-140.25
	Dx	Serum	121.00	98.00-140.50
	Dx	Urine	0.13	0.00-0.43
	MC	Plasma	15.20	11.43-18.70
	MC	Serum	16.20	12.48-19.80
	MC	Urine	0.07	0.00-0.34
	PsO	Plasma	0.00	0.00-0.23
	PsO	Serum	0.14	0.00-0.46
	PsO	Urine	0.00	0.00-0.17
**9**	Dx	Plasma	20.58	16.25-24.33
	Dx	Serum	18.98	10.78-24.65
	MC	Plasma	4.53	2.18-6.58
	PsO	Plasma	0.42	0.00-0.92
	PsO	Urine	0.00	0.00-0.23
**10**	MC	Plasma	1.93	0.14-3.23
	PrO	Plasma	1.29	0.34-1.96
	PrO	Urine	0.00	0.00-0.27
	PsO	Plasma	0.07	0.00-0.36

Time points as follows: *Dx*, diagnosis; *MC*, midchemotherapy; *PrO*, preoperative; *PsO*, postoperative; *EOT*, end of treatment. Wild-type *TP53* from cell free DNA is not shown.

**Figure 1:**
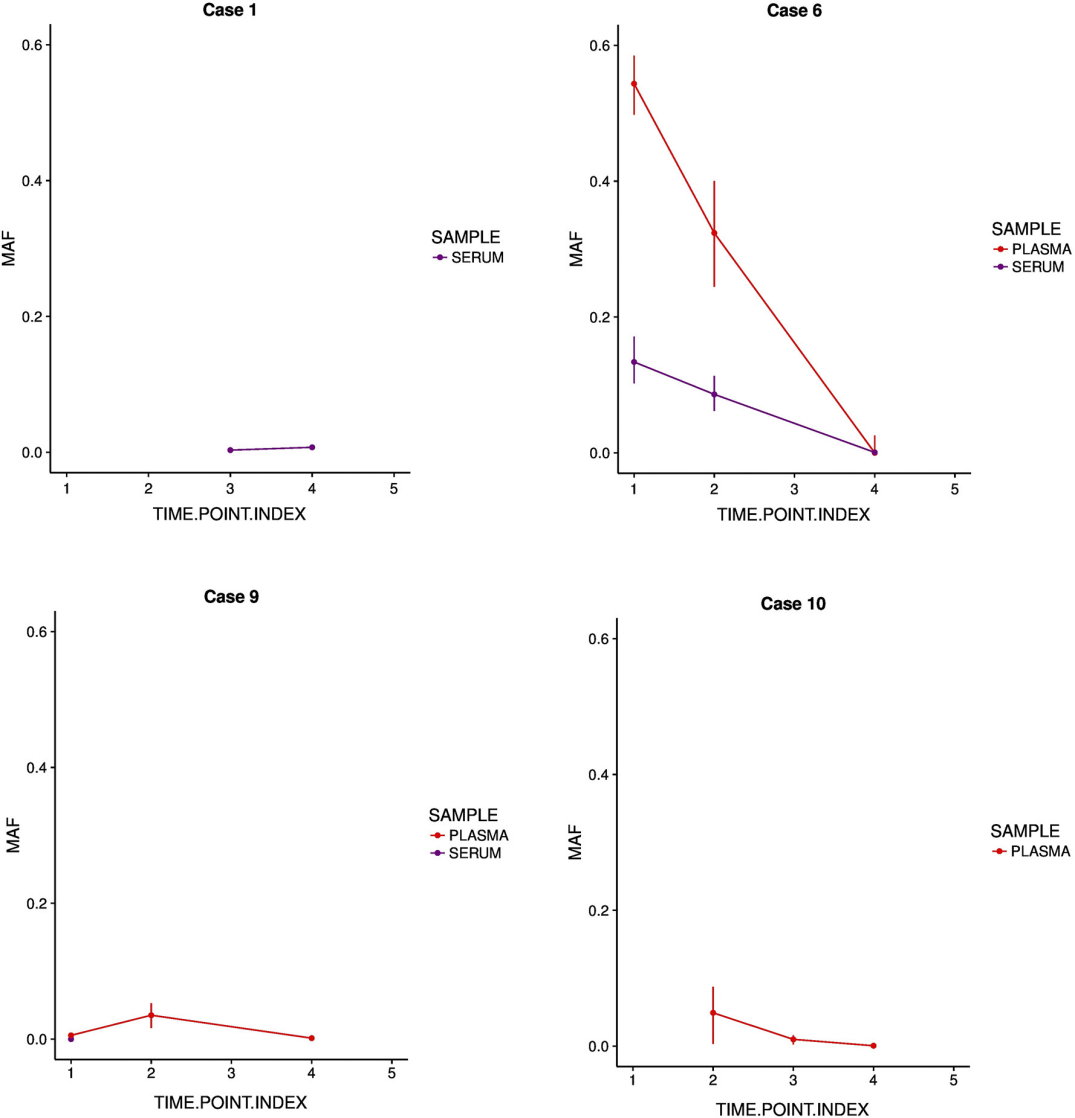
MAF from serum and plasma for each case varies during treatment. Time points as follows: 1, diagnosis; 2, mid-chemotherapy; 3, pre-operative; 4, post-operative; and 5, end of treatment. MAF is calculated as mutant allele concentration divided by total concentration. Error bars represent the range of MAFs produced within the confidence intervals of the mutant and wild-type allele concentrations. Urine ctDNA is not shown.

In three out of four cases, MAF decreased following nephrectomy (Figure 1). In case 1, ctDNA concentration increased from 0.44 (0.05-0.90) copies/μl to 6.63 (4.93-8.15) copies/μl after nephrectomy. We cannot rule out that the high concentration postoperatively is due to manipulation of the tumor during surgery. However, owing to the very short half-life of ctDNA, this effect is likely to be negligible. This patient has stage III disease and relapsed 339 days postoperatively. In case 1 and case 10, wild-type *TP53* (as assessed by cell-free DNA levels) increased postoperatively.

**Supplementary Figure 1:**
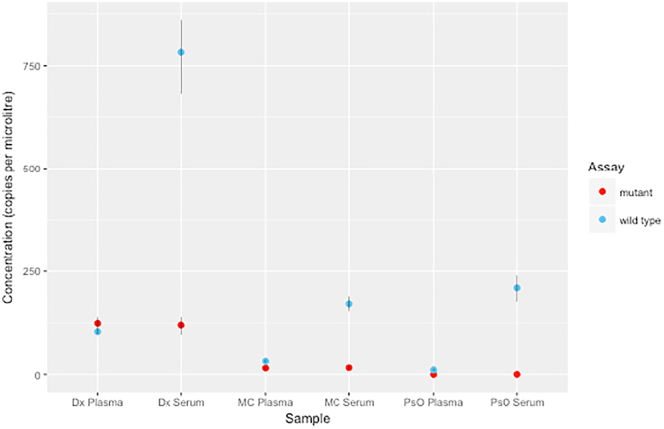
Contamination from germline cell-free DNA in serum versus plasma for case 6 at three different time points throughout treatment. Time points as follows: *Dx*, diagnosis; *MC*, midchemotherapy; *PsO*, postoperative.

Genomic contamination, thought to be from lysed blood cells, is greater in serum than in plasma (Supplementary Figure 1). Concentration of ctDNA was significantly lower in 200 μl of urine supernatant than those from the same volume of plasma or serum (Table 2). The concentration of mutant ctDNA extracted from 1.8 ml of urine from case 1 postoperatively was 35.50 (32.25-38.75) copies/μl.

**Supplementary Figure 2:**
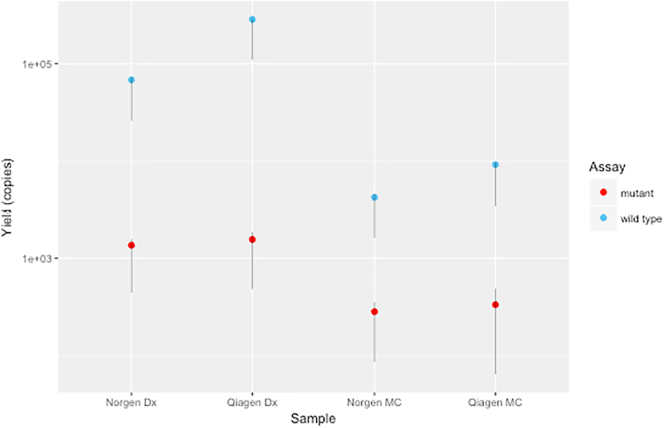
Comparison of ctDNA yield extracted by Qiagen and Norgen from plasma for case 9. Time points as follows: *Dx*, diagnosis; *MC*, midchemotherapy; *PrO*, preoperative; *PsO*, postoperative.

Comparison of extraction kits (Qiagen circulating nucleic acid kit versus Norgen Plasma/Serum Cell-free Circulating DNA Purification Mini Kit) was undertaken in two cases with four samples overall. Results are consistent with a previous study and demonstrate comparable ctDNA yields (Supplementary Figure 2) [17].

## Discussion

Our explorative study demonstrates that collection and analysis of bodily fluids taken from children with WT are achievable. Further, mutant *TP53* is detectable in ctDNA from plasma and serum in patients with DAWT, though not from the same volume of urine.

Although somatic *TP53* mutations are infrequent in childhood cancer, pediatric pan-cancer analyses have identified *TP53* as the most frequent pathogenic constitutional mutation [18], [19], [20]. There are over 200 SNPs in the gene, with some of these variants likely predisposing to cancer [21]. In adults, somatic alterations in *TP53* are the commonest genetic event, with six frequently observed hotspot residues [22]. Two of the mutations we identified (p.R248Q and p.R273C) align to data from 25,902 patients showing that these sites are the two most common loci for *TP53* mutations [23], [24]. Regarding the amino acid residue R248, recurrent *TP53* mutations in WT have been observed at this locus [24]. Three mutations (p.R248Q, p.R273C, and p.H179L) are considered to be gain of function (GOF) and are associated with a highly aggressive phenotype, while p.R337C is thought to be a nonfunctional mutant [25].

Mutations in *TP53* are seemingly associated with a higher risk of relapse and death for children with advanced-stage anaplastic WT [3], [4]. Originally thought to be pathognomonic for DAWT, a recent analysis of 84 fatal cases found that 26% of nonanaplastic tumors harbored mutations in *TP53* [26]. There are no studies that have assessed circulating *TP53* status in nonanaplastic fatal tumors, but this is clearly an avenue that requires exploration. With a mutational spectrum from GOF to nonfunctional mutants, any decisions to intensify therapy for children with mutant *TP53* are likely to be complex. As DAWT is relatively uncommon, with only around 150 cases annually in Europe and America, collaboration is vital in addressing these clinical questions.

A further possible merit in assessment of *TP53* status is targeted therapy [27]. Early-phase trials in adult relapsed/refractory solid and hematological malignancies are ongoing to test both *TP53* recombinant adenoviral human gene therapy as well as inhibitors of MDM2/MDMX, negative regulators of p53 [28]. Interestingly, some patients with GOF mutants have demonstrated response to MDM2/MDMX targeted therapy, although there remains the theoretical risk that these inhibitors could increase levels of mutant *TP53* and drive cancer progression [28].

Our study was conducted as a proof of principle and hence was carried out on a small number of patients (*n* = 4) and their associated ctDNA samples (*n* = 21). Although, to date, 446 patients have been recruited to the IMPORT study, only 8%-10% are expected to have anaplastic WT, and only a minority of these patients had both tumor sampling and samples from which to extract ctDNA (*n* = 10). A further limitation is that samples were not taken at identical time points for each patient. This is due to the intrinsic difficulties associated with venous and urine sampling in young children, complicated by multicenter sample collection, each having variable resources. Due to genomic contamination, plasma is preferential to serum in applications such as NGS where the mutant may be obscured by the wild-type allele. For clinical utility, urinary ctDNA would be preferable to plasma as sampling is minimally invasive. We were able to detect mutant *TP53* in ctDNA from 1.8 ml of urine collected from case 1. A recent analysis in children with *PIK3CA*-related overgrowth spectrum syndrome found that *PIK3CA* variants were detectable by ddPCR in 3 ml of urine from five of eight patients [29]. Similar results have been demonstrated in adult urological malignancies including renal cell carcinoma where, like WT, cells may not be in direct contact with urine [30].

At the time of experimental design, to our knowledge, there were no published data describing either DNA yields or MAFs in ctDNA from patients with early-stage WT. As such, ddPCR was chosen for its limit of detection. However, in its current form, ddPCR is unlikely to be of clinical use for determining *TP53* status in WT, as each patient requires a mutation-specific assay. The issues with a personalized assay involve unknown performance of bespoke primer sets and cost. It was decided not to design a mutation-specific assay for case 2 as this process would have lacked the experimental validation of commercially produced sets. A targeted NGS panel would permit analysis of the entire *TP53* gene, and we have shown that MAFs in this cohort are large enough to allow the detection of circulating *TP53* mutations. Genomic DNA contamination can be reduced with size selection during library preparation, as ctDNA is fragmented [31]. A further merit in ctDNA is that it captures different tumor clones and could be used in lieu of tumor multisampling. Though ctDNA from a larger cohort of patients with WT needs to be characterized to ensure all driver subclonal mutations are detectable. In patients with recurrent hotspot mutations in the microRNA processing genes (*DROSHA*, *DGCR8*) or the transcription factors *SIX1*/*SIX2*, ddPCR may one day play a role in monitoring via serial liquid biopsies [32], [33].

WT is a genetically heterogeneous group, and the current list of WT-related cancer genes is fast approaching 40 [32], [33], [34]. However, few of these genes are linked as strongly with clinical outcome as mutant *TP53* [6], [7], [26]. In the future, NGS-based methods could be employed to screen blood/urine collected at diagnosis for *TP53* mutations, allowing early intensification of preoperative chemotherapy for this poorer prognostic group. In addition, ctDNA could be monitored throughout treatment and used for surveillance post end of treatment. The primary aim of our investigation was as a feasibility study, and we have shown that mutant circulating *TP53* is detectable in children with WT, even in low-burden disease and regardless of whether the underlying mutation is subclonal or homogeneous.

The following are the supplementary data related to this article.
